# The Molecular Basis of the Effect of Temperature on the Structure and Function of SARS-CoV-2 Spike Protein

**DOI:** 10.3389/fmolb.2022.794960

**Published:** 2022-03-25

**Authors:** Faez Iqbal Khan, Kevin A. Lobb, Dakun Lai

**Affiliations:** ^1^ Department of Biological Sciences, School of Science, Xi’an Jiaotong-Liverpool University, Suzhou, Jiangsu, China; ^2^ School of Electronic Science and Engineering, University of Electronic Science and Technology of China, Chengdu, China; ^3^ Department of Chemistry, Rhodes University, Grahamstown, South Africa

**Keywords:** SARS-CoV-2, COVID-19, spike protein, MD simulations, Gibbs free energy

## Abstract

The remarkable rise of the current COVID-19 pandemic to every part of the globe has raised key concerns for the current public healthcare system. The spike (S) protein of SARS-CoV-2 shows an important part in the cell membrane fusion and receptor recognition. It is a key target for vaccine production. Several researchers studied the nature of this protein under various environmental conditions. In this work, we applied molecular modeling and extensive molecular dynamics simulation approaches at 0°C (273.15 K), 20°C (293.15 K), 40°C (313.15 K), and 60°C (333.15 K) to study the detailed conformational alterations in the SARS-CoV-2 S protein. Our aim is to understand the influence of temperatures on the structure, function, and dynamics of the S protein of SARS-CoV-2. The structural deviations, and atomic and residual fluctuations were least at low (0°C) and high (60°C) temperature. Even the internal residues of the SARS-CoV-2 S protein are not accessible to solvent at high temperature. Furthermore, there was no unfolding of SARS-CoV-2 spike S reported at higher temperature. The most stable conformations of the SARS-CoV-2 S protein were reported at 20°C, but the free energy minimum region of the SARS-CoV-2 S protein was sharper at 40°C than other temperatures. Our findings revealed that higher temperatures have little or no influence on the stability and folding of the SARS-CoV-2 S protein.

## Introduction

The outbreaks of Severe Acute Respiratory Syndrome CoV 1 (SARS-CoV-1), Middle-East Respiratory Syndrome CoV (MERS-CoV), and Severe Acute Respiratory Syndrome CoV 2 (SARS-CoV-2) were caused by zoonotic viruses in 2003, 2012, and 2019–2020 with a fatality ratio of 10%, 35%, and 5%, respectively (Lee et al., 2003; Cheng et al., 2007; Zaki et al., 2012; de Groot et al., 2013; Reusken et al., 2013; Rothan and Byrareddy, 2020). The International Virus Classification Commission (ICTV) termed this 2019 novel CoV as SARS-CoV-2 (Chen et al., 2020; Zhu et al., 2020). SARS-CoV-2 virus spread from humans to humans, and animals to humans (Khan et al., 2020a; Khan et al., 2021a). The COVID-19-infected patient develops mild to moderate symptoms and recovers. Some patients have serious symptoms such as atypical pneumonia and chest pain (Huang et al., 2020a; Cheung et al., 2020; Lu et al., 2020; Rothan and Byrareddy, 2020). The phenomenal spread of the current COVID-19 pandemic to every part of the sphere has raised key concerns for the healthcare system. To combat this pandemic, the researchers are using all possible approaches and practices to inhibit the synthesis of crucial non-structural viral proteins, inhibit the viral replicase enzyme, inhibit the formation of viral RNA, prevent the self-assembly of viruses, or boost the human immune response against the virus.

The Spike (S) protein of SARS-CoV-2 performs a vital part in the cell membrane fusion and receptor recognition. It has two subunits such as S1 and S2. A receptor-binding domain (RBD) is present on the S1 subunit. The RBD recognizes and attaches to the host receptor angiotensin-converting enzyme 2 (ACE-2). The membrane fusion (MF) is facilitated by the S2 subunit by making 6 helical bundles through two heptad repeat (HR) domains (Huang et al., 2020b). The S protein has a size of 180–200 kDa. It has an extracellular N-terminal, a transmembrane (TM) attached to the membrane, and small intracellular C-terminal domains.

The S proteins are covered with polysaccharide for camouflage and escaping the host immune system in the course of entry (Huang et al., 2020b). The S protein of SARS-CoV-2 contains 1,273 amino acid residues. It contains a signal peptide (1–13 amino acids), an S1 subunit (14–685 residues), and an S2 subunit (686–1,273 residues). The S1 subunit has 14–305 N-terminal domain amino acids and 319–541 RBD amino acids. The S2 subunit has 788–806 fusion peptide (FP) amino acids, 912–984 HR1 amino acids, 1,163–1,213 HR2 amino acids, 1,213–1,237 TM domain amino acids, and 1,237–1,273 cytoplasm domain amino acids (Xia et al., 2020). The SARS-CoV-2 S protein lives as a sedentary precursor in native state. During the viral contagion, the proteases from target cells trigger the S protein by slicing it into two different subunits (Bertram et al., 2013), which is needed for triggering the MF domain after entry of virus into the target cells.

The SARS-CoV-2 S protein is an important target for vaccine production. In this work, we applied molecular modeling and molecular dynamics simulations approaches at 0°C (273.15 K), 20°C (293.15 K), 40°C (313.15 K), and 60°C (333.15 K) to study the detailed conformational variations in the SARS-CoV-2 S protein. It is worth noting that, experimentally, in terms of information on inactivation of viruses, data in the range 40–60°C are essential (Bertrand et al., 2012). Our findings revealed that higher temperatures have little or no influence on the SARS-CoV-2 S protein. We found that the structural deviations, atomic, and residual fluctuations were least at low (0°C) and high (60°C) temperature. The solvent accessible area plot indicated that the internal residues of SARS-CoV-2 spike protein are not exposed to solvent at high temperature. The secondary structure scheme indicated that there was no such denaturation of the SARS-CoV-2 S protein at higher temperature. The most stable conformations of the SARS-CoV-2 S protein was found at 20°C, but the free energy state region of SARS-CoV-2 spike protein was sharper at 40°C than other temperatures.

## Materials and Methods

### Structure Modeling of the SARS-CoV-2 S Protein

The structures of S protein of SARS-CoV-2 (PDB: 6vsb) were taken from PDB (Wrapp et al., 2020). The missing atoms in the structure of S protein were modeled using MODELLER (Webb and Sali, 2016). The complete protocols are stated in preceding publications (Khan et al., 2015; Khan et al., 2016c; Khan et al., 2017a; Khan et al., 2017b; Khan et al., 2021d). Structure analysis was performed using PDBsum (Laskowski et al., 2018) and numerous modules of MD simulations. PyMOL was used for visualization and drawing structure.

### MD Simulations

MD simulations were achieved on SARS-CoV-2 spike protein at 0°C (273.15 K), 20°C (293.15 K), 40°C (313.15 K), and 60°C (333.15 K) *via* GROMACS 2018.2 (Van Der Spoel et al., 2005) using a standard protocol (Khan et al., 2020b; Khan et al., 2020c; Khan et al., 2021b). Na^+^ and Cl^−^ ions were supplemented to neutralize the system. Absolute production phase of 100 ns was attained at 0°C (273.15 K), 20°C (293.15 K), 40°C (313.15 K), and 60°C (333.15 K). The complete MD simulation procedure is cited in prior publications (Khan et al., 2016a; Khan et al., 2016b; Durrani et al., 2020; Hassan et al., 2020; Qausain et al., 2020).

### Essential Dynamics

ED was obtained for the SARS-CoV-2 S protein at 0°C (273.15 K), 20°C (293.15 K), 40°C (313.15 K), and 60°C (333.15 K). It is estimated as:

$$C_{ij}=<\left(r_{i}-\langle r_{i}\rangle \right)\times \left(r_{j}-\langle r_{j}\rangle \right)\left(i,j=1,2,3,\ldots 3N\right).$$ (1)

*r*_*i*_ denotes the coordinate, *i*th Cα atom, *N* signifies the Cα atoms, and <*r*_*i*_ > indicates time average over all configurations (Khan et al., 2020d).

### Gibbs Free Energy Landscape

GFE landscape can suggest conformational variations in the SARS-CoV-2 S protein at 0°C (273.15 K), 20°C (293.15 K), 40°C (313.15 K), and 60°C (333.15 K) (Khan et al., 2016c). The GFE landscape was projected on PC1 and PC2.

$$G_{\left(PC1,PC2\right)}=-k_{B}T\text{In}P_{\left(PC1,PC2\right)}.$$ (2)

*k*_B_, *T*, and *P*_(*PC1, PC2*)_ denote Boltzmann constant, temperature, and normalized joint probability distribution for SARS-CoV-2 spike protein at 0°C (273.15 K), 20°C (293.15 K), 40°C (313.15 K), and 60°C (333.15 K) respectively.

## Results and Discussion

### Structure Analysis of Spike Protein

**FIGURE 1:**
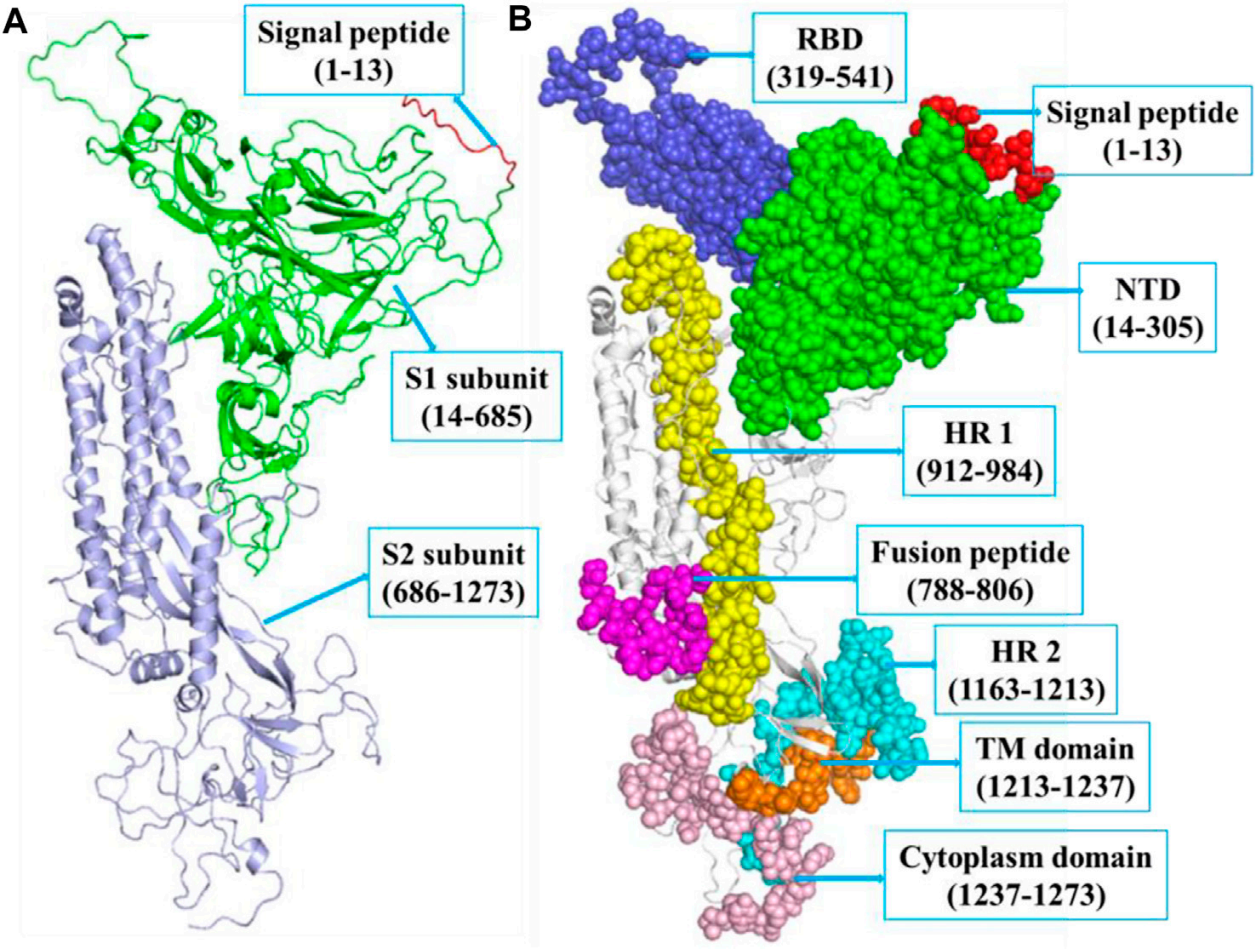
**(A)** The structure of SARS-CoV-2 spike protein indicating SP (red, 1–13 *aa*), S1 (green, 14–685 *aa*), and S2 (blue, 686–1,273 *aa*), respectively. **(B)** The S1 subunit includes NTD domain (green, 14–305 *aa*) and RBD (blue, 319–541 *aa*). The S2 subunit includes FP (magenta, 788–806 *aa*), hepta-peptide repeat sequence 1 (yellow, 912–984 *aa*), hepta-peptide repeat sequence 2 (cyan, 1,163–1,213 *aa*), TM domain (orange, 1,213–1,237 *aa*), and cytoplasm domain (pink, 1,237–1,273 *aa*), respectively.

The SARS-CoV-2 spike protein comprises N-terminal, TM, and C-terminal segments (Bosch et al., 2003). It consists of a signal peptide (1–13 residues at the N-terminal), an S1 subunit (14–685 amino acid residues), and an S2 subunit (686–1,273 amino acid residues). The S1 is accountable for receptor attachment, and S2 is accountable for membrane fusion. The S1 subunit contains 14–305 NTD residues and 319–541 RBD residues. The S2 contains 788–806 FP amino acid residues, 912–984 HR1 residues, 1,163–1,213 HR2 residues, 1,213–1,237 TM domain residues, and 1,237–1,273 cytoplasm domain residues (Huang et al., 2020b). The residues that participated in strand, α-helix, and 3–10 helix formations are 271 (28.3%), 190 (19.8%), and 23 (2.4%), respectively. The structure of spike protein includes 18 β-hairpins, 13 β-sheets, 52 β-strands, 18 β-bulges, 29 helix-helix interactions, 22 helices, 16 γ-turns, 76 β-turns, and 12 disulfides (Figure 1).

### Structural Deviations

**FIGURE 2:**
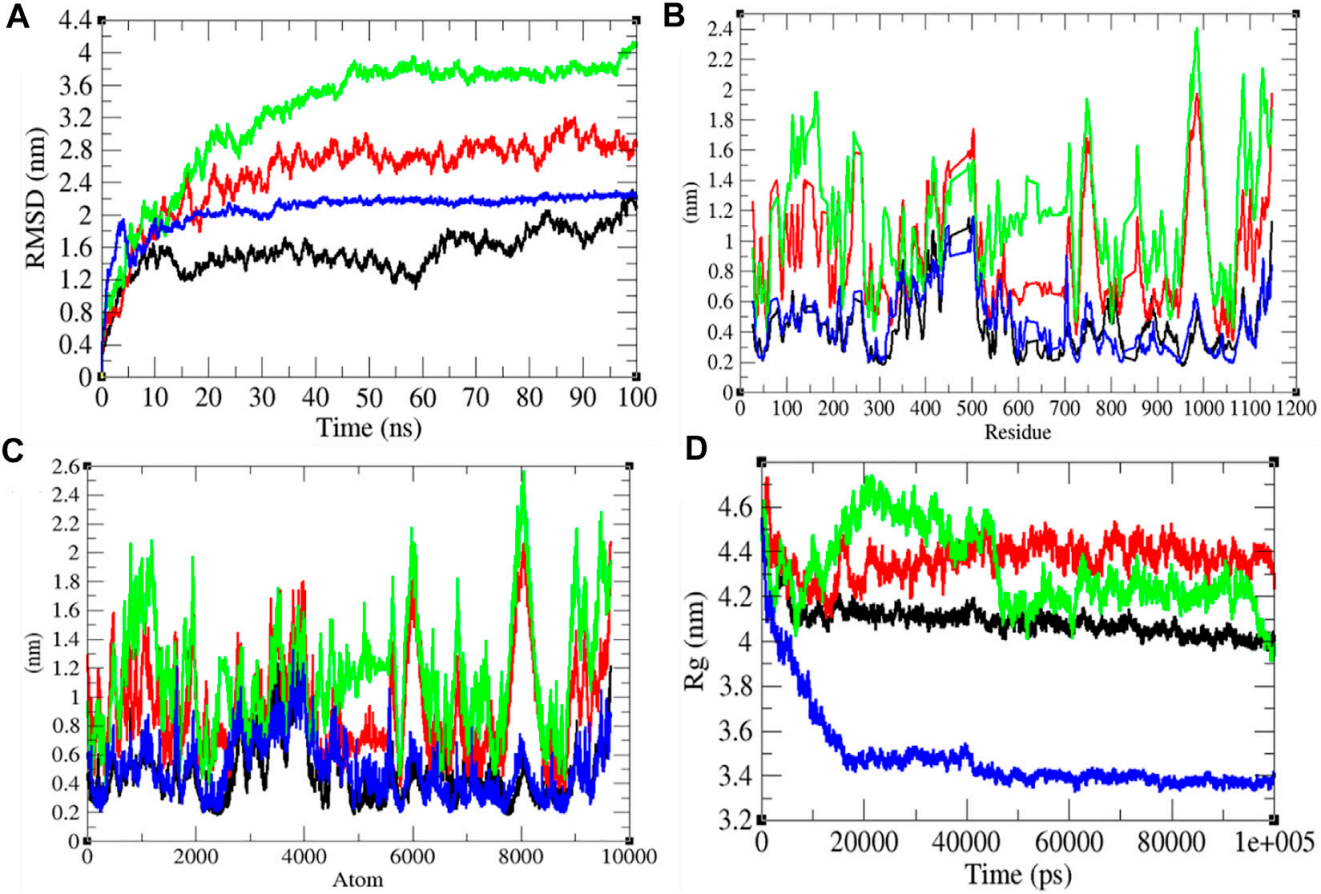
Structural dynamics. **(A)** RMSD plot for the SARS-CoV-2 S protein vs. time. **(B)** RMSF vs. residues. **(C)** RMSF vs. residues. **(D)** *R*_g_ plot vs. time. The values calculated at 0°C (black), 20°C (red), 40°C (green), and 60°C (blue), respectively.

To investigate the structural dynamics of the SARS-CoV-2 S protein, the RMSD, RMSF, and the *R*_g_ were considered throughout 100-ns MD simulations at 0°C (273.15 K), 20°C (293.15 K), 40°C (313.15 K), and 60°C (333.15 K), respectively (Kuzmanic and Zagrovic, 2010). The mean RMSD values of the SARS-CoV-2 S protein at 0, 20, 40, and 60°C were estimated to be 1.53, 2.51, 3.26, and 2.23 nm, respectively (Figure 2). It has been estimated that RMSD values, and residual and atomic fluctuations increase from 0 to 40°C. It attained a low structural deviation equilibrium at 60°C. The SARS-CoV-2 S protein is least deviated at low (0°C) and high (60°C) temperature. The average radius of gyration (*R*_g_) values for the SARS-CoV-2 S protein at 0, 20, 40, and 60°C was found to be 4.09, 4.37, 4.32, and 3.48 nm, respectively. The *R*_g_ is described as the allotment of atoms of a molecule around its axis. The calculation of *R*_g_ is a significant indicator that is broadly used in calculating the structural activity. At different temperatures, there is a conformational change in the SARS-CoV-2 S protein that changes the radius of gyration. It was estimated that the SARS-CoV-2 S protein is tightly packed at 60°C. At 20°C–40°C, it shows high fluctuations throughout the time scale.

### Solvent Accessible Surface Area

**FIGURE 3:**
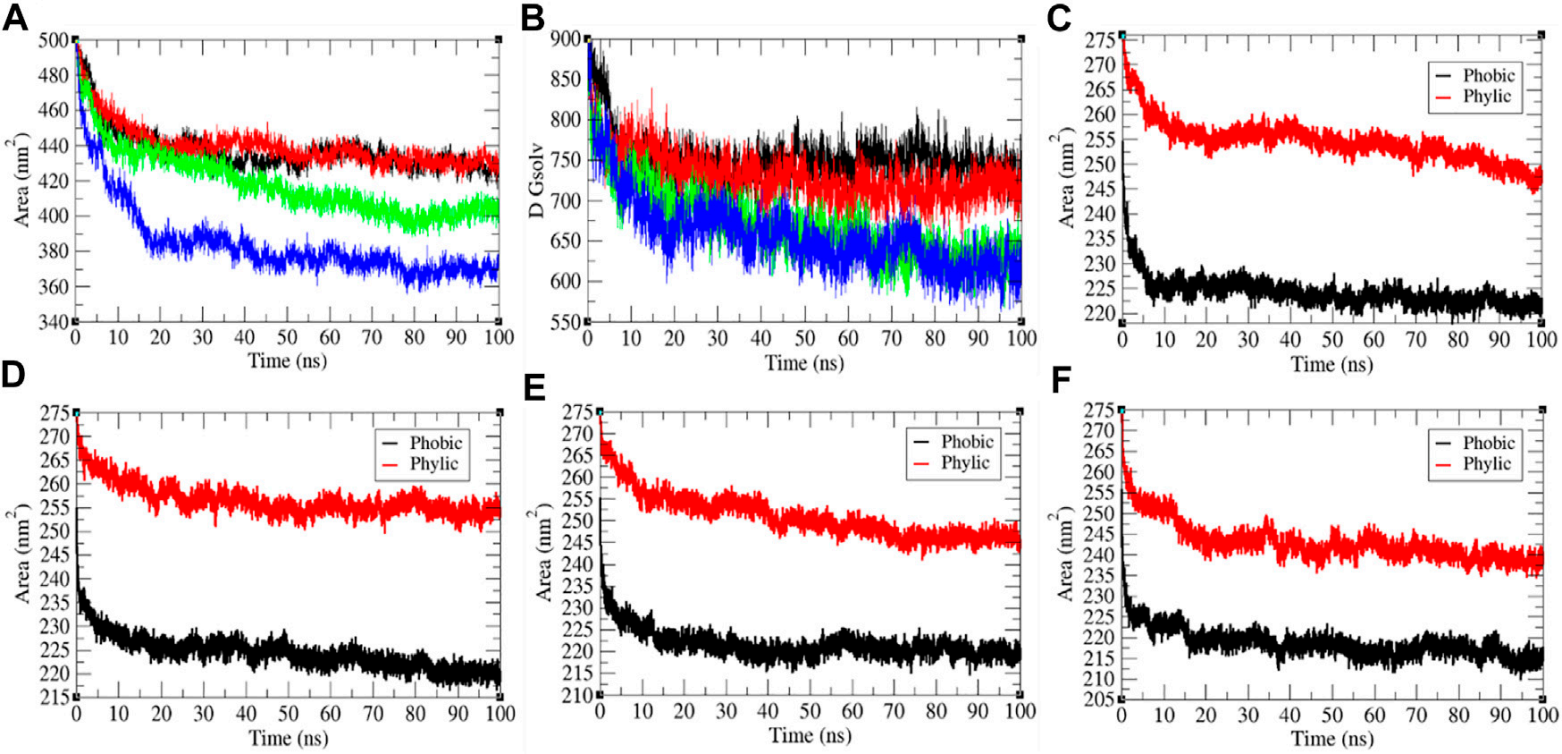
The solvent accessible surface area of the SARS-CoV-2 S protein. **(A)** SASA vs. time. **(B)** Free energy of solvation vs. time. The color codes have the same meaning as described in Figure 2. It was resolved into hydrophobic and hydrophilic regions for the SARS-CoV-2 S protein at **(C)** 0°C, **(D)** 20°C, **(E)** 40°C, and **(F)** 60°C, respectively.

It has been assumed as a significant element in molecular stability and folding analysis. The average solvent accessible surface area values for the SARS-CoV-2 S protein at 0, 20, 40, and 60°C were found to be 437.71, 439.92, 418.90, and 384.66 nm^2^, respectively (Figure 3). The solvation energy for the SARS-CoV-2 S protein at 0, 20, 40, and 60°C was found to be 752.14, 730.93, 668.86, and 657.95 kJ/mol/nm^2^, respectively. An increase in temperature from 0 to 20°C has not much effect on SASA of the SARS-CoV-2 S protein. At 40°C–60°C, the solvent accessible surface area of the SARS-CoV-2 S protein continuously decreases. This specifies that the internal residues of the SARS-CoV-2 S protein are not exposed to solvent at high temperature. This might be due to stability and compactness of the SARS-CoV-2 S protein at higher temperature. The solvation energy refers to the free-energy change during the simulations. The solvation free energy is also less at higher temperature. The solvent accessible surface area was further divided into hydrophobic and hydrophilic regions. The hydrophobic regions for the SARS-CoV-2 S protein at 0, 20, 40, and 60°C were found to be 224.22, 224.34, 221.61, and 218.62 nm^2^, respectively. The hydrophilic regions for the SARS-CoV-2 S protein at 0, 20, 40, and 60°C were 254.52, 256.53, 250.9, and 243.08 nm^2^, respectively. Both hydrophobic and hydrophilic regions are sparingly accessible to solvent at higher temperatures.

### Secondary Structure Analysis

**FIGURE 4:**
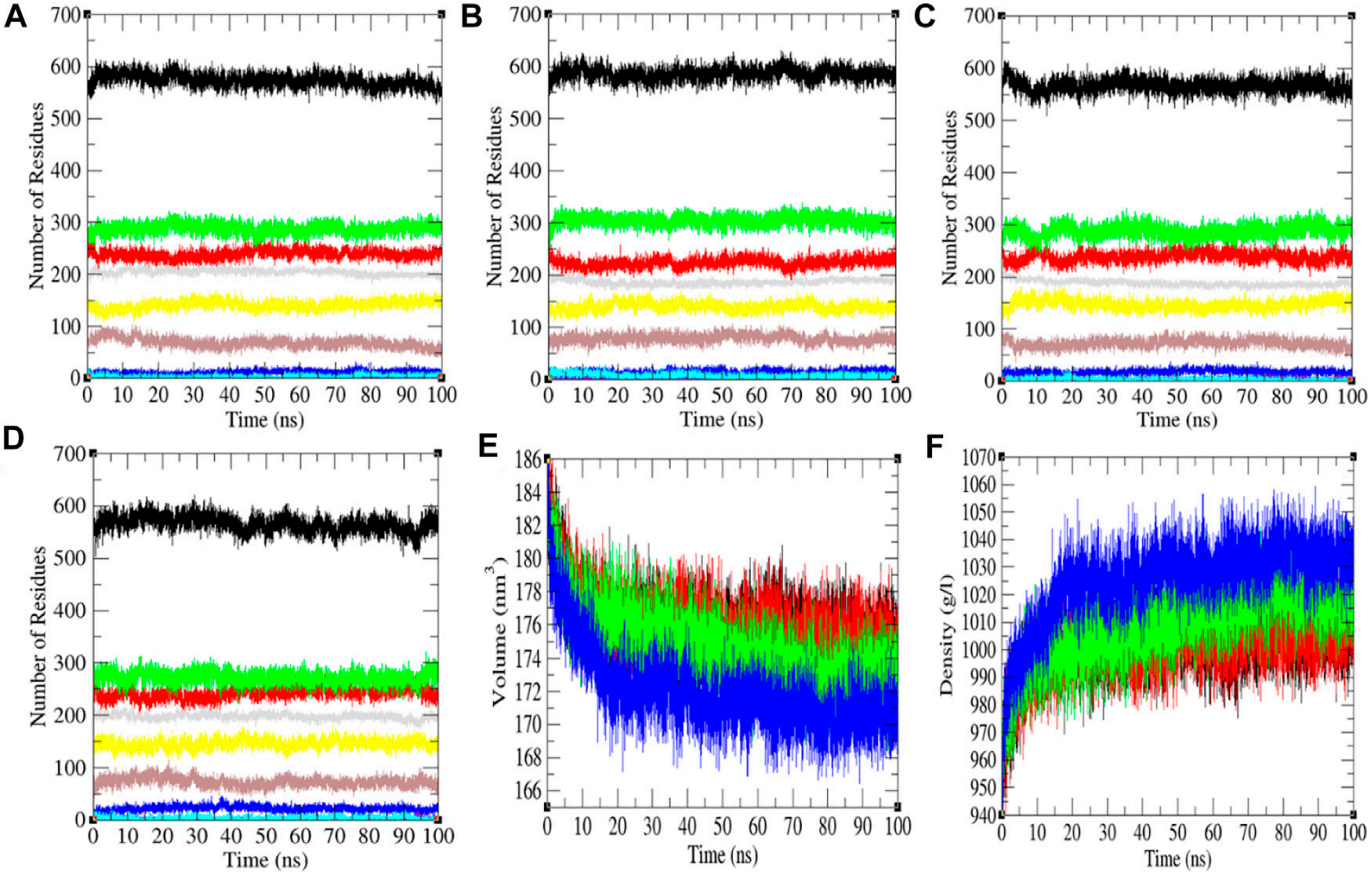
The secondary structure conformations. The secondary structure plot of the SARS-CoV-2 S protein at **(A)** 0°C, **(B)** 20°C, **(C)** 40°C, and **(D)** 60°C, respectively. **(E)** Volume and **(F)** density of SARS-CoV-2 spike protein. The color codes have the same meaning as described in Figure 2.

The secondary structure in the SARS-CoV-2 S protein was analyzed at each period at 0, 20, 40, and 60°C (Table 1). The mean residues involved in the assembly of the SARS-CoV-2 S protein at 0, 20, 40, and 60°C were found to be 60%, 61%, 59%, and 59%, respectively (Figure 4). There was no such unfolding of the SARS-CoV-2 S protein reported at higher temperature from this analysis. The β-sheet of the SARS-CoV-2 S protein slightly unfolds from 30 to 28% at 60°C, while the α-helix (21%) remained unchanged at 60°C. The most stable conformation of the SARS-CoV-2 S protein was found at 20°C. Furthermore, we calculated the volume and density of the SARS-CoV-2 S protein at 0, 20, 40, and 60°C, respectively. Moreover, the volume of the SARS-CoV-2 S protein was found to be 176.02, 176.04, 174.71, and 172.03 nm^3^ at 0, 20, 40, and 60°C, respectively, while the density of the SARS-CoV-2 S protein was calculated to be 1,001.84 g/L, 1,001.72 g/L, 1,009.39 g/L, and 1,025.18 g/L at 0, 20, 40, and 60°C, respectively. The volume of the SARS-CoV-2 S protein slightly decreases and density increases at higher temperature. This might be due to different structure conformations at higher temperatures.

**TABLE 1:** Percentage of residues in SARS-CoV-2 spike protein at 0, 20, 40, and 60°C contributed in mean structure development.

Temperature (°C)	Secondary structure (SS %)							
	Structure^a^	Coil	β-sheet	β-bridge	Bend	Turn	α-helix	3_10_-helix
0	60	25	30	1	15	7	21	0
20	61	23	32	2	15	8	20	1
40	59	25	30	2	15	8	20	0
60	59	25	28	2	15	8	21	0

a Structure = α-helix + β-sheet + β-bridge + Turn.

### Hydrogen Bonding and the Mean Square Displacement

**FIGURE 5:**
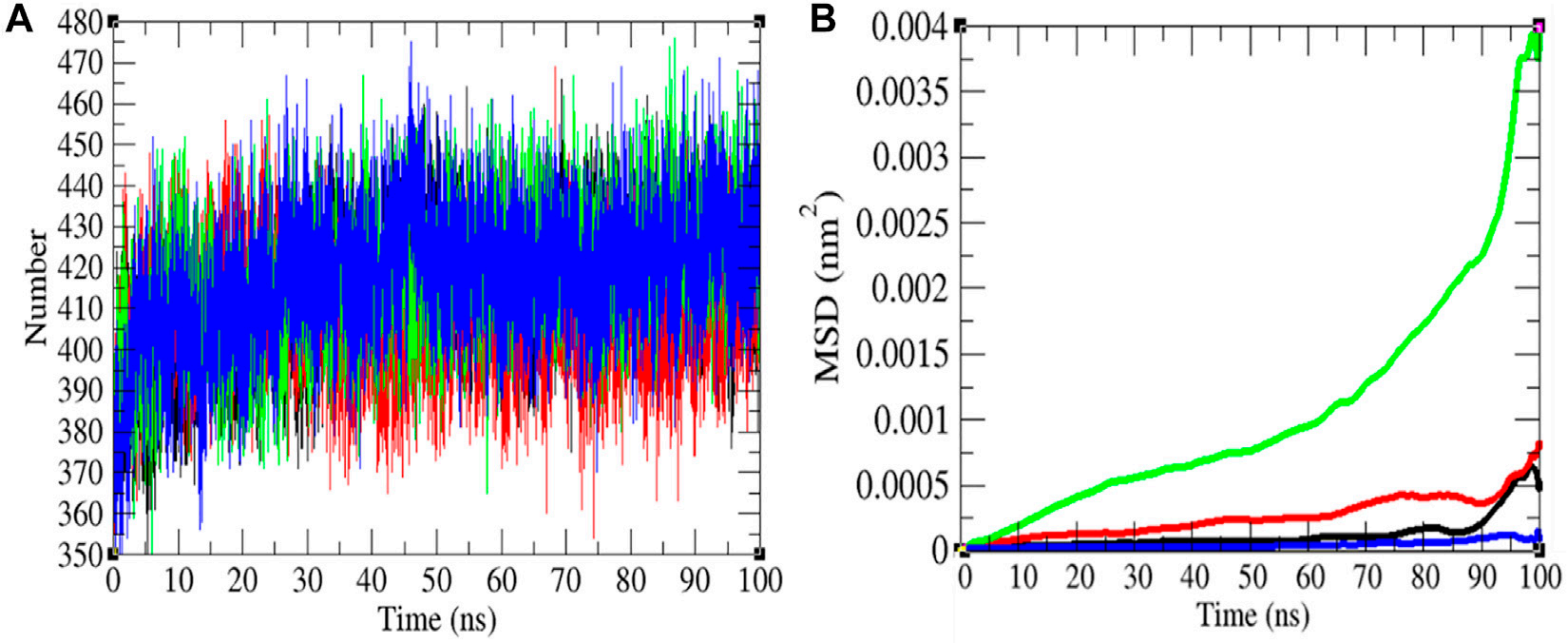
Hydrogen bonds and MSD. **(A)** The H-bond estimation between M-S chains of the SARS-CoV-2 S protein was calculated. The color codes have the same meaning as described in Figure 2. **(B)** The MSD of the SARS-CoV-2 S protein at 0°C (black), 20°C (red), 40°C (green), and 60°C (blue), respectively.

The H-bond is a noteworthy element in stabilizing the molecule. It was estimated between the main chain and side chains (M-S) of the SARS-CoV-2 S protein at 0, 20, 40, and 60°C, respectively. The mean H-bonds between M-S chains of the SARS-CoV-2 S protein were found to be 413.43, 407.89, 417.99, and 417.51 at 0, 20, 40, and 60°C, respectively (Figure 5). The strength of hydrogen bonds becomes stronger at 40–60°C. There is no sign of denaturation at higher temperatures. Furthermore, the mean square displacement (MSD) of atoms from a set of original positions of the SARS-CoV-2 S protein at 0, 20, 40, and 60°C was computed. The displacement of atoms from a set of initial positions of the SARS-CoV-2 S protein was estimated to be higher at 40°C only. In short, higher temperature has not much impact on unfolding and denaturation of the SARS-CoV-2 S protein.

### Principal Component Analysis

**FIGURE 6:**
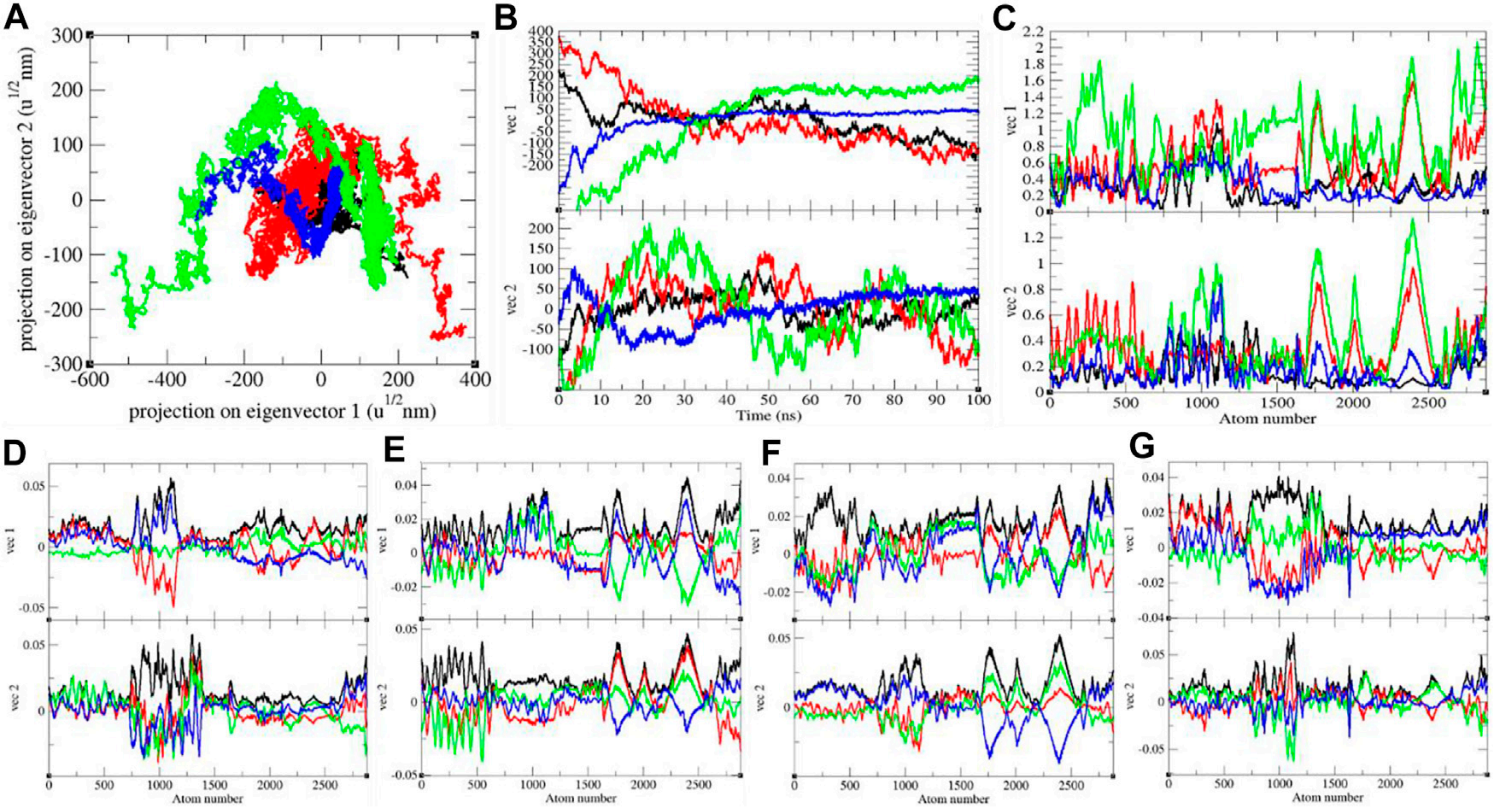
**(A)** The 2D projection and **(B)** projections of trajectories of SARS-CoV-2 spike protein. **(C)** Eigen RMSF. The color codes have the same meaning as described in Figure 2. The Eigen components were calculated for SARS-CoV-2 spike protein at **(D)** 0°C, **(E)** 20°C, **(F)** 40°C, and **(G)** 60°C, respectively.

It shows global expansion of the SARS-CoV-2 S protein at 0, 20, 40, and 60°C. It estimates mean atomic motions of the SARS-CoV-2 S protein at 0, 20, 40, and 60°C. The eigenvalues were 8,360.61, 33,665.60, 53,083.50, and 8,911.24 nm^2^ for the SARS-CoV-2 S protein at 0, 20, 40, and 60°C, respectively. It was higher at 20–40°C (Figure 6). The average atomic motions in the SARS-CoV-2 S protein was highest at 40°C. The atomic motions are also related to activity in case of protein molecules. It can be assumed that at low and high environmental temperatures, the atomic motions and activity of the SARS-CoV-2 S protein are low.

### GFE Landscape

**FIGURE 7:**
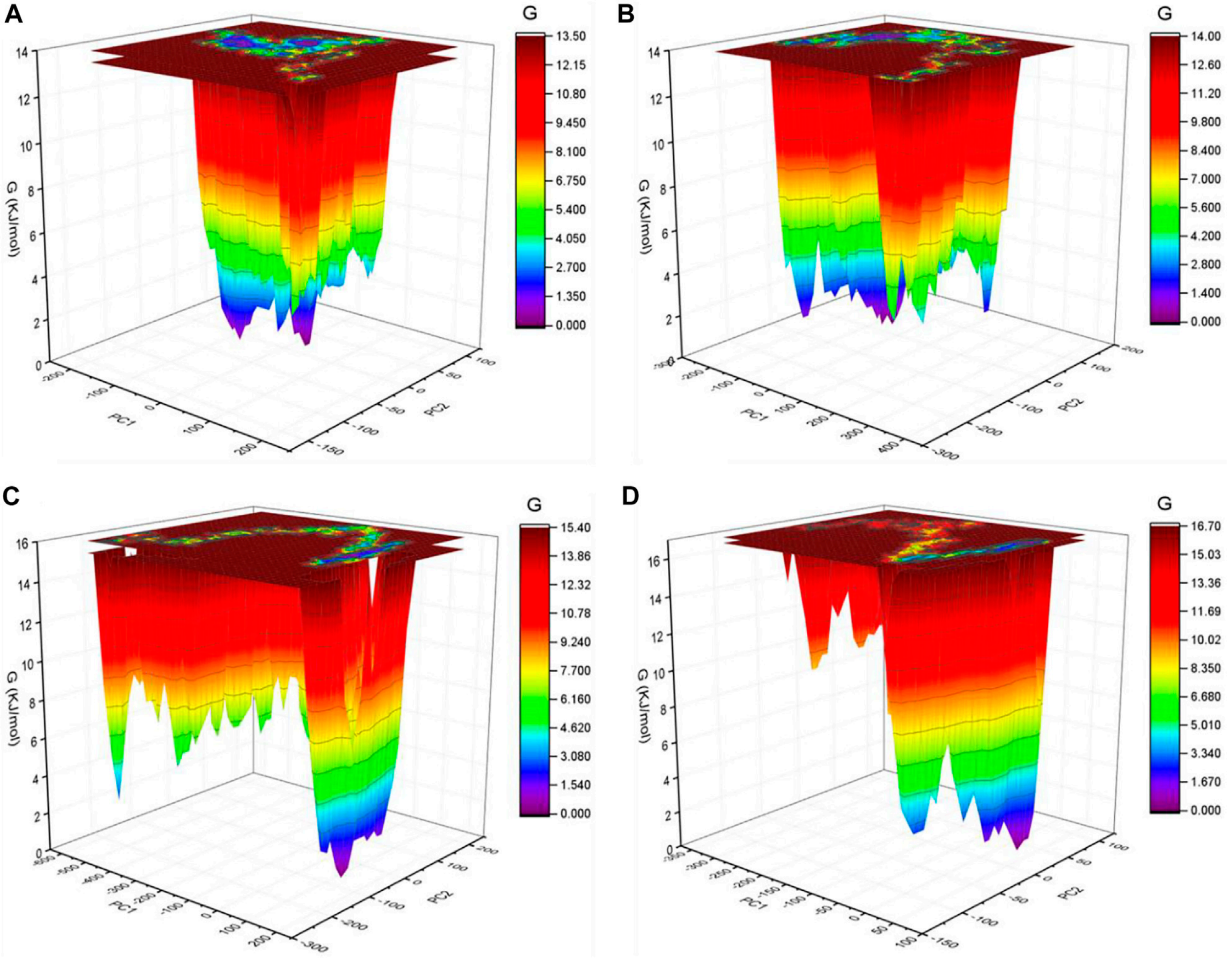
GFE landscape. The GFE landscape plot achieved for SARS-CoV-2 spike protein at **(A)** 0°C, **(B)** 20°C, **(C)** 40°C, and **(D)** 60°C, respectively.

The GFE landscape exhibited diverse forms for the SARS-CoV-2 S protein at 0, 20, 40, and 60°C (Figure 7). Every atomic pair covariance displays diverse frameworks in respective events. The GFE patterns are relatively similar with minor changes at 0–20°C and 40–60°C. The following GFE curve with reflective blue shade implies lower energy state. Extra blue regions describe shifts in the molecular conformation lagged by the thermodynamically new favorable areas. The GFE state in the global energy minimum section of the SARS-CoV-2 S protein at 40°C is sharper than other temperatures. This indicates that temperature slightly affects the GFE patterns in the case of the SARS-CoV-2 spike protein. The GFE landscape suggests that the temperature slightly affects the atomic motions of SARS-CoV-2 spike protein. The denaturation was not reported from the secondary structure analysis. The potential energy and the enthalpy were also calculated during the course of simulations. The potential energy was found to be −10,713,734.28 kJ/mol, −10,434,949.84 kJ/mol, −10,161,602.16 kJ/mol, and −9,893,572.08 kJ/mol at 0, 20, 40, and 60°C, respectively. The enthalpy was found to be −9,100,901.71 kJ/mol, −8,704,052.14 kJ/mol, −8,312,630.26 kJ/mol, and −7,926,537.36 kJ/mol at 0, 20, 40, and 60°C, respectively.

Recently, several inhibitors and their mode of action have been demonstrated (Khan et al., 2021c; Rani et al., 2021). Edwards et al. found that the spike protein samples kept at diverse temperatures did not show any considerable denaturation, while they observed an increase in upper molecular weight bands in a sample that was kept at 37°C (Edwards et al., 2021). Kumar et al. imitated SARS-CoV-2 by polymer beads covered with the S protein of SARS-CoV-2 to investigate the effect of different temperatures on attachment of virus-imitating nano-spheres to lung tissues incubated at 33 and 37°C. They found that the existence of the RBD of S protein controlled the binding by Calu-3 airway epithelial tissues. They also found that there was no temperature correlation to binding of BSA-coated nano-spheres. Additionally, the 4–40°C temperature had no influence on S-RBD-ACE-2 ligand–receptor, and the negligible effect on the S-RBD protein structure (up to 40°C) was reported. The protein denaturation occurred at 51°C. Their outcomes suggested that 4–40°C temperature has a slight influence on SARS-CoV-2 and ACE-2 contact (Kumar et al., 2021). Zhou et al. performed MD simulations at 36–40°C to prove SARS-CoV-2 and ACE2 binding. They found that it was less stable under 40°C than under 37°C, and reduced infection rate at higher temperature (Zhou et al., 2021). Martí et al. also performed MD simulations at 298 K (24.85°C), 310 K (36.85°C), 324 K (50.85°C), 338 K (64.85°C), 358 K (84.85°C), and 373 K (99.85°C), respectively. They suggested that temperature brings structural and conformational variations in the S1 subunit and affects the RBD. Nevertheless, the influence of temperature up to 373 K was not adequate to cause a noteworthy alteration of the S protein of SARS-CoV-2 (Martí et al., 2021). Our results also suggested that the temperature has the least effect on the structure conformations of S protein of SARS-CoV-2.

## Conclusion

Previously, we published several articles based on finding potential inhibitors of SARS-CoV-2. In the present work, we focused on the SARS-CoV-2 S protein as it performs a vital part in the cell membrane fusion and receptor recognition. Researchers have demonstrated the nature of the S protein of SARS-CoV-2 on diverse environmental conditions. We applied molecular modeling and extensive molecular dynamics simulations approaches at different temperatures to investigate the structural conformational of SARS-CoV-2 spike protein. There are several hypotheses proposed regarding the temperature dependence of the COVID-19 transmission. We concluded that temperature has no effect or has the least effect on the structure conformations of S protein of SARS-CoV-2. Minor changes were reported in the structure and thermodynamic properties that are mentioned in this paper.

## Data Availability

The datasets presented in this study can be found in online repositories. The names of the repository/repositories and accession number(s) can be found in the article/Supplementary Material.
